# Room4Birth - the effect of an adaptable birthing room on labour and birth outcomes for nulliparous women at term with spontaneous labour start: study protocol for a randomised controlled superiority trial in Sweden

**DOI:** 10.1186/s13063-019-3765-x

**Published:** 2019-11-19

**Authors:** Marie Berg, Lisa Goldkuhl, Christina Nilsson, Helle Wijk, Hanna Gyllensten, Göran Lindahl, Kerstin Uvnäs Moberg, Cecily Begley

**Affiliations:** 1 Institute of Health and Care Sciences, Sahlgrenska Academy, Gothenburg University, Box 457, SE-405 30 Gothenburg, Sweden; 2 Department of Obstetrics and Gynecology, Sahlgrenska University Hospital, SE-416 50 Gothenburg, Sweden; 3 Faculty of Caring Science, Work Life and Social Welfare, University of Borås, SE-501 90 Borås, Sweden; 4 Quality and Patient Safety Unit, Sahlgrenska University Hospital of Gothenburg, SE-413 45 Gothenburg, Sweden; 5 Centre for Healthcare Architecture , CVA, Chalmers University of Technology, SE-412 96 Gothenburg, Sweden; 6 Centre for Person-Centred Care (GPCC), University of Gothenburg, Box 457, SE-405 30 Gothenburg, Sweden; 7 Building Design, Architecture and Civil Engineering, Chalmers University of Technology, SE 412 96 Gothenburg, Sweden; 8 University of Agriculture (SLU), Uppsala, Sweden; 9 School of Nursing and Midwifery, Trinity College Dublin, The University of Dublin, 24 D’Olier St, Dublin 2, Ireland

**Keywords:** Birth environment, Birthing room, Person-centred care, Labour, Randomised controlled trial

## Abstract

**Background:**

An important prerequisite for optimal healthcare is a secure, safe and comfortable environment. There is little research on how the physical design of birthing rooms affects labour, birth, childbirth experiences and birthing costs. This protocol outlines the design of a randomised controlled superiority trial (RCT) measuring and comparing effects and experiences of two types of birthing rooms, conducted in one labour ward in Sweden.

**Methods/design:**

Following ethics approval, a study design was developed and tested for feasibility in a pilot study, which led to some important improvements for conducting the study. The main RCT started January 2019 and includes nulliparous women presenting to the labour ward in active, spontaneous labour and who understand either Swedish, Arabic, Somali or English. Those who consent are randomised on a 1:1 ratio to receive care either in a regular room (control group) or in a newly built birthing room designed with a person-centred approach and physical aspects (such as light, silencer, media installation offering programmed nature scenes with sound, bathtub, birth support tools) that are changeable according to a woman’s wishes (intervention group). The primary efficacy endpoint is a composite score of four outcomes: no use of oxytocin for augmentation of labour; spontaneous vaginal births (i.e. no vaginal instrumental birth or caesarean section); normal postpartum blood loss (i.e. bleeding < 1000 ml); and a positive overall childbirth experience (7–10 on a scale of 1–10). To detect a difference in the composite score of 8% between the groups we need 1274 study participants (power of 80% with significance level 0.05). Secondary outcomes include: the four variables in the primary outcome; other physical outcomes of labour and birth; women’s self-reported experiences (the birthing room, childbirth, fear of childbirth, health-related quality of life); and measurement of costs in relation to the hospital stay for mother and neonate. Additionally, an ethnographic study with participant observations will be conducted in both types of birthing rooms.

**Discussion:**

The findings aim to guide the design of birthing rooms that contribute to optimal quality of hospital-based maternity care.

**Trial registration:**

ClinicalTrials.gov NCT03948815. Registered 13 May 2019—retrospectively registered.

## Background

### Introduction to current practice: childbirth care in Sweden

In Sweden, healthcare at birth is offered only at hospitals. The aim of this care is to promote a physiologically normal birth, i.e. where labour starts spontaneously and ends in a vaginal non-instrumental birth, where mothers and babies are healthy and the mothers have a positive childbirth experience [1]. The proportion of physiologically normal births has, however, gradually decreased. By 2018, 21.5% of all births in Sweden were medically induced compared to 7.1% in 1991, and 17.2% of women gave birth through caesarean section (CS), compared to 10.9% in 1991 [2]. Also by 2018, 53.2% of first-time mothers with spontaneous labour received oxytocin infusion for labour augmentation [3]. This high level of intervention in otherwise normal labour warrants increased costs due to increased demands on personnel during the birth itself, prolonged hospital stay, and more frequent readmission in the postnatal period [4]. For example it has been shown to be cost-effective in women with one previous CS to have a vaginal birth on the next pregnancy compared to having a repeat CS [5]. Although a recent trial in the United States has shown a decrease in CS in low risk nulliparous women randomised to have induction of labour at 39 weeks gestation [6], the population comprised women who were predominantly of Hispanic, Asian or African ethnicity (56%), were unemployed (49%) and had obesity (52%). As that population is usually at greater risk of pregnancy and labour complications, and differs considerably from the Swedish population, maternity care in Sweden has not changed practice as a result.

### Existing knowledge on the effect of healthcare environment on health

A secure, safe and comfortable environment is an important prerequisite for providing optimal healthcare [7]. The environment can, however, be experienced differently by patients/clients. A technically sound healthcare facility, for example, provides a sense of security for some people while for others it can create feelings of alienation and a reduced sense of self-determination [8–10]. Thus, for optimal safety, health and efficiency, the care environment should be adaptable enough to meet the unique needs of patients and their companions [11]. Physical design aspects [12] such as single rooms, good ventilation [13, 14], windows [14], conditions that promote orientation and distraction [14, 15], a view of or access to nature, real or artificial [13, 14], and ergonomic furniture [14] can all have positive health effects. Psychological dimensions [12] such as staff with a willingness to help, promotion of integrity, conveying a sense of security and trust through more person-centred care also improve patient/client health [16, 17]. The following aspects contributing to a supportive care environment where it is possible to experience ease have been identified in a tentative theoretical construction: experiencing a genuine welcome; recognising oneself in the space provided; creating and maintaining social relations; experiencing a willingness to be served and cared for; and experiencing safety [16].

### The mechanism of the effect of environment on birth

Labour and birth are innate, biological, instinctive processes that have always been, and still are, linked to certain risks [18, 19]. Therefore, mammalian mothers have instinctively always chosen to give birth in an environment perceived as safe, secure and private [18, 20]. When a woman in labour arrives at the hospital, factors such as loud noise, light and a strange, unfriendly environment increase the activity of some parts of the brain cortex and amygdala that signal danger, and the body’s stress and defence systems are activated. This leads to inhibition of oxytocin release and/or sympathetic nervous system activity may increase. As a result, labour contractions can become too strong and painful, or even cease [18, 20–23], leading to medical intervention [19], such as that seen in the high rate of augmentation of labour with synthetic oxytocin [3]. The opposite also applies; if the cortex and amygdala perceive the environment as safe, harmless, friendly and inviting, this leads to physical and mental relaxation and to decreased fear and stress responses. This leads to more effective labour contractions and to good uterine blood circulation, which positively affect the progress of birth [22, 24], increase oxygenation of the foetus, and prevent postpartum haemorrhage. Touch, warmth and closeness also promote the release of oxytocin and decrease stress levels [25].

Women will remember and be affected by their labour and birth events throughout their lives [26, 27]. Women who feel safe and who can open up to the flow and rhythm of labour can be strengthened [28, 29], but the experience can also leave negative impressions [30]. Non-empathetic treatment by healthcare professionals and threatening or over-medicalised birthing environments can leave negative impressions [31]. A negative experience of birth can cause ill-health in women, such as posttraumatic stress disorder, depression, persistent intense fear of childbirth [31–34], delayed subsequent pregnancy [35, 36] and demands for future operative birth [36, 37].

### Existing knowledge on the effect of healthcare environment on birth

The healthcare environment in relation to birth is inadequately studied. A systematic review by our research group (unpublished), with searches of ten databases in December 2016 and August 2017, showed that birthing rooms that provide comfort using controllable stimuli can provide distraction from labour pain, comfort, control and safety [38] and have positive effects on duration of labour and pain intensity [39, 40]. Sense of familiarity has a positive impact on feelings of control, ownership of space and nest-building behaviours of women and their birthing companions [41–43], while feelings of unfamiliarity distract women when giving birth [44]. These findings clearly suggest that design aspects of a birthing room can influence the outcomes of giving birth and affect women’s decisions about future mode of birth [45]. Our review shows that there is a lack of scientific studies with sufficient quality on the effect of the birthing room itself.

A pilot RCT in Canada found that it was feasible to perform an RCT comparing a more flexible birthing room to a regular one, and that fewer women who had given birth in the flexible birthing room received synthetic oxytocin for augmentation of contractions. The authors recommended a full RCT [46], but at the time of commencement of our planned study no full-scale RCT had been reported. We have found two ongoing RCTs comparing care in a specially designed birthing room with care in a regular room. A single-centre study in Denmark examines whether a birthing room specially designed and decorated to minimise stress has an impact on birth outcomes and the birth experience of the woman and her partner. One of the existing birthing rooms has been rebuilt using a wooden material, home-like non-clinical furniture and a wall projector with nature scenes combined with music and nature sounds. The RCT plans to include 680 nulliparous women at term randomly allocated to either the rebuilt birthing room or the regular birthing room. The primary outcome is augmentation of labour with oxytocin [47]. An ongoing multicenter study in 12 labour wards in Germany will evaluate if a redesigned birthing room that facilitates mobility and upright positioning, coping with pain and personal comfort will result in a higher probability of a vaginal birth in hospital. The environment in the intervention room has been re-conceptualised with special features and equipment, such as a bed hidden from sight by a screen or curtain, a mattress on the floor, foam elements for support, a bean bag and posters depicting upright positions, and a monitor showing nature films with natural sound and music. The study will comprise 3800 women and has vaginal birth as the primary outcome [48].

## Methods/design

Based on the lack of knowledge that was identified concerning the effect and influence of birthing room on the woman and her baby, and the likelihood that maternity wards in some countries will be in need of either reconstruction or new construction of labour wards, we identified the need to conduct a randomised controlled trial (RCT).

This study protocol (version 1, 19th June 2019) is described in accordance with the SPIRIT checklist (Standard Protocol Items: Recommendations for Interventional Trials) [49]. For details see Additional file 1. All major changes to the protocol (e.g. changes to eligibility criteria, outcomes, analyses) will be notified to the trial registry, ethics review board, investigators and participants.

### Aim, hypothesis and study design

The overall purpose of this project is to extend the evidence-based knowledge on the design of birthing rooms and their influence on labour, birth and childbirth experiences in nulliparous women in active, spontaneous labour at ≥ 37 gestational weeks.

The hypothesis has been inspired by and built on the mechanism of the effect of environment on birth [18–25], our own, unpublished systematic literature review, interviews with women some days after having given birth [50], aspects pinpointed by health care professionals at the study hospital and women from a user council (the agency Födelsehuset) information from the pilot study conducted in Canada [46], and from the ongoing study in Denmark [47].

We hypothesise that the physical design aspects of a birthing room have effects on the users (woman, companion and staff), and that users interact with the room and make the space their own by using it and giving meaning to it, making it their place. We also hypothesise that a more adaptable person-centred birthing room may facilitate and enable healthy labour and birth processes and outcomes by reducing stress and facilitating the release of endogenous oxytocin. This will reduce the need for augmentation of labour with exogenous (synthetic) oxytocin, increase the likelihood of a vaginal, spontaneous birth, reduce pathological haemorrhage, increase the occurrence of a positive childbirth experience and reduce fear of childbirth and of giving birth again, without any adverse effects on maternal and neonatal outcomes. We further hypothesise that the added costs of creating a more adaptable person-centred birthing room are compensated for by reduced costs for medical interventions and complications during the hospital stay. Using the more adaptable person-centred birthing room may also give an all-around better experience of the birth process for the woman’s companion and improved job satisfaction for staff, but this has to be, and is planned to be, studied in future projects.

Since the experience and influence of the healthcare environment is personal and complex, its effects have to be studied with objectively measurable methods as well as qualitative methods [7].

Our study is a randomised, controlled unblinded superiority trial (RCT), with two parallel groups comparing the effects of two types of birthing rooms. Care in a regular birthing room will be compared with care in an adaptable birthing room with a person-centred approach (‘new room’). In this new birthing room, the woman can change physical aspects according to her own choices, thus creating an environment with a sense of safety, integrity and familiarity. Various outcomes of labour and birth, women’s self-reported experiences of the birthing room, childbirth and quality of life are measured. Furthermore, costs are compared between the care in the regular room and the new room. An additional ethnographic study will explore the influence and meaning of the birthing rooms (regular rooms and new room) on and for women giving birth.

### Intervention

The study takes place at one of three labour wards at Sahlgrenska University Hospital (SUH), Gothenburg in the west of Sweden. It takes into account a newly renovated birthing room, new technology and education of staff to make best use of the updated facilities.

The labour ward serves women at ≥ 34 weeks of pregnancy. In 2018 the labour ward had 4237 births, where 1372 (32.4%) of the women were nulliparous with a single foetus in cephalic presentation at ≥ 37 weeks with spontaneous start of labour.

All birthing rooms are fully equipped with all necessary medico-technical devices. One of the regular birthing rooms has been reconstructed into a person-centred adaptable birthing room for the intervention group. This room provides the woman with more choices to change it according to her preferences. The main differences between the new room and the regular rooms are described in Table 1.

**Table 1:** Main differences between the new room and the regular room

Content	New room	Regular birthing room
Size	23.8 m^2^	19 m^2^
Entrance hall	Yes, 3 m^2^	No
Toilet with shower	Yes	Yes
Bathtub	Yes	No
Window, opening	Yes, hidden if media installation in use	Yes
Lighting	Yes, several options with dimming function	Yes, several options, no dimming
Silencer	Yes, a 40-mm suspended sound absorber in the ceiling	No
Media installation	Yes, installation covers two walls, including the window. Offers choice of programmed nature scenes with light, sound effects and music	No
Birthing bed, ordinary	Yes, covered with homelike bedspread	Yes, no bedspread
Medico technical equipment	Yes, hidden behind a wood-panel wall, which is rolled up when necessary	Yes, fully visible
Rounded corners on furniture	Yes, some	No
Sofa	Yes, can be converted to an extra bed for companion	No
Chair for companion	Yes, designed for comfort, adjustable height	Yes, ordinary model
Mirror	Yes	Yes
Pilates ball	Yes	No
Birth support rope	Yes	No
Cabinet for personal belongings	Yes, with ability to recharge electronic devices such as mobile phone	No

### Recruitment and randomisation of participants

Eligible participants are women ≥ 18 years of age that are classified as “Robson 1”, i.e. nulliparous women at ≥ 37 gestational weeks, with a single live foetus in cephalic presentation, and in spontaneous labour [51]. They should understand either Swedish, English, Arabic or Somali or have access to an interpreter when necessary. When arriving at the labour ward they should be in the active phase of labour as defined in Sweden at the time of study, i.e. two of these three criteria fulfilled: spontaneous rupture of membranes; two or three painful contractions in 10 min; cervix dilated > 3–4 cm or effaced and open ≥ 1 cm. Women with induced labour, planned caesarean section, multiple gestation or in the latent phase of labour will be excluded from the study.

General information about the study is provided to nulliparous women in the antenatal maternity clinics, where most women who give birth at the hospital are cared for. No details about the different types of rooms are given, including no photos. This is to prevent expectations on the part of women or companions who might develop a strong desire to give birth in the specially designed room and then be disappointed if they are allocated to a regular birthing room. It is clarified in the information leaflet that access to the new room is only available through the study and no woman allocated to a regular room can transfer into the new room at any stage.

All nulliparous women arriving at the labour ward are received in either a regular room or a so-called assessment room. The midwife clarifies if the woman is in active labour or not by checking the woman’s contractions, rupture of membranes, pain status, bleeding, fetal position by abdominal palpation and, in most cases, cervical status by vaginal examination. Auscultation of fetal heartbeat with a Pinard’s stetoscope and recording of fetal heartbeat for 20–30 min with cardiotocography is also performed.

If both types of birthing rooms are vacant (i.e. the new room and one of the seven regular rooms), a woman fulfilling the inclusion criteria, and her companion, will be informed about the study orally and by written information, and will be invited to participate. The supplied information comprises:

- There are two types of birthing rooms that will be tested in the study, a specially designed room that has increased potential to be adapted to personal wishes and needs, and a regular birthing room.
- It is only the physical design that differs between the two rooms—the specially designed room will have the same level of medical safety and technology as the regular room.
- The responsible staff will explain the functions that are available in the room to which the woman will be allocated.

Since the midwife often needs time to clarify if the woman is in active labour or not, randomisation directly at time of arrival is usually not possible. Women who are admitted when the new room or one of the regular birthing rooms are not available will not be included in the study. Women who choose not to participate in the study will not be included in the study. The protocol is applied in exactly the same way for everyone; thus, all women are treated equally.

A woman who wants to participate signs a written consent and is randomised to care in either the new room (intervention group) or to care in one of the regular rooms (control group). The randomisation system is managed by an independent statistician at an agency who has prepared an allocation list based on randomly generated numbers and placed the designated allocation in sequentially numbered, sealed envelopes kept in a study folder only available for staff members at the labour ward. To minimise the risk of detection bias, the woman will at randomisation be provided with the unique four-figured ID code printed on the sealed envelope. The midwife opens the next sealed envelope containing details of the allocation and informs the woman and her partner that they have been allocated to either the new room or the regular room. Blinding of either the attending staff or the participants is not possible. Midwives are not aware of the randomisation sequence and the envelopes are opaque. The independent statistician ensures that the randomisation procedure is followed.

**Fig. 1:**
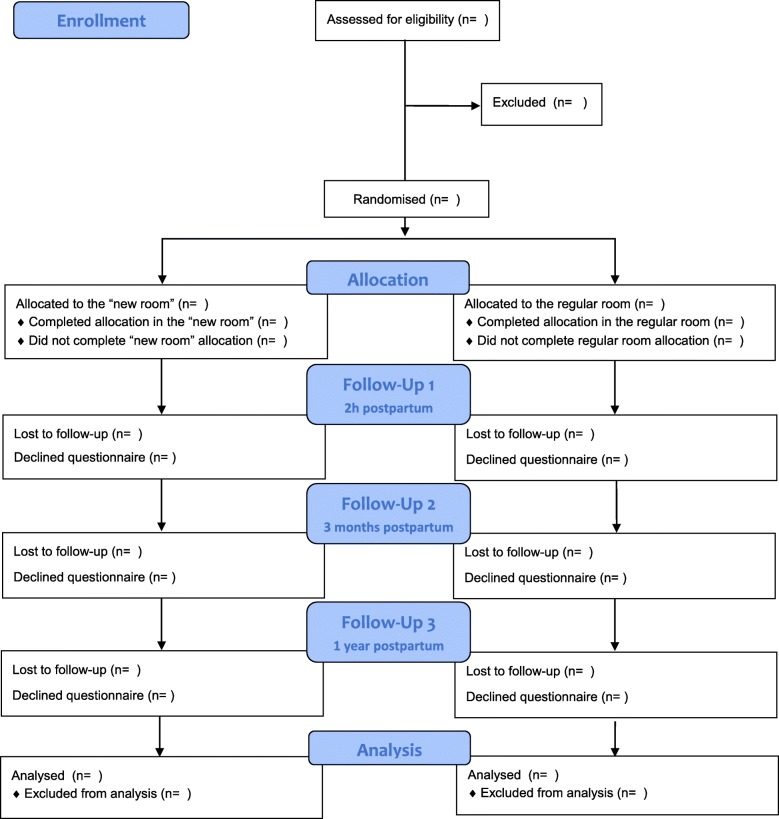
Flowchart of the Room4Birth randomised controlled trial

The responsible midwife and other staff will follow the woman to the randomised room and provide care following the same care guidelines in both types of room. The enrollment and flow of the RCT is illustrated in Fig. 1.

Any woman wishing to withdraw from the study, from either group, will be withdrawn immediately. However, without any persuasion from the researchers, she will be asked if she is willing for her data to remain in the database. If she agrees, her data will remain and be analysed with the rest of her allocated group’s data (i.e. intention to treat); if she disagrees, her data will be deleted. If a woman randomised to the new room wants to withdraw from the study at any time, she will be transferred and cared for in a regular room (i.e. standard care in that hospital). If a woman randomised to a regular room wants to withdraw from the study at any time, she will continue to be cared for in that room (i.e. standard care in that hospital).

### Pilot testing

The study was pilot tested during December 2018 to test the study routines, identify and eliminate potential obstacles and simplify the implementation of the study. Before the start of the pilot study the staff at the labour ward received instruction on how to use the facilities in the room and the room was regularly used for the staff to feel comfortable within the environment. After that, nine women fulfilling the inclusion criteria were included in the pilot study. The pilot test showed that the study procedure was feasible; some information sheets were revised to be more understandable and a new checklist was developed to facilitate identification of eligible women. Furthermore, the inclusion criteria further specified that the included woman must be in active labour at enrollment and not spend several hours in a regular room before randomisation since this could affect the experience of the birthing environment negatively.

### Data collection and outcome measures

The primary efficacy endpoint is a composite score measured before discharge from the hospital and compared between the two groups. To ensure holistic data for assessing the effect of the room on labour and birth outcome, both medical and experiential outcomes are included in the score. The composite score is 1 if all the following four parts of the composite variable are fulfilled and 0 otherwise:

- No use of synthetic oxytocin for augmentation of labour
- Spontaneous vaginal births (i.e. no vaginal instrumental birth or CS)
- Normal postpartum haemorrhage (i.e. bleeding < 1000 ml)
- A positive overall childbirth experience: 7–10 on a Likert scale with anchor 1 = bad, 10 = good

The secondary efficacy outcomes that will be measured and compared between the two groups are: all four primary variables, body mass index (BMI), gestational week at birth, seeking help for fear of childbirth, mental illness treatment, rupture of membranes, amniotomy, use of synthetic oxytocin for augmentation of labour (duration and highest dose), duration of labour, duration of active labour, use of bathtub, use of epidural anaesthesia, maternal fever during labour, duration of pushing stage, position of woman for vaginal birth, episiotomy, mode of birth (vaginal spontanoeus, vacuum extraction and CS), indication for instrumental vaginal birth and CS, cervical tear, perineal tears divided in grades, postpartum haemorrhage (> 1000 ml), manual removal of placenta, stillbirth, neonatal death during hospital stay, Apgar score at 5 min (1 to 10, and grouped < 4 and < 7), umbilical cord blood samples at birth, sex of newborn, birth weight, skin to skin in the first hour, breastfeeding within first 2 hours, admission to the neonatal unit, and length of mother’s hospital stay. Self-reported experiences and outcomes will be measured 2 h after birth, after discharge, after 3 and 12 months with specific questions concerning experience of the room, childbirth ecperience as a whole, Childbirth Experience Questionnaire (CEQ) [52], Fear of Birth Scale (FOBS) [53], fear of giving birth again [54] and health-related quality of life using the EuroQol 5 Dimension (EQ-5D) health state questionnaire [55]. If the woman does not answer the 3- or 12-month questionnaires she will get up to three reminders once a week, the first two reminders by email followed by one phone text message. Using such techniques in our previous studies resulted in response rates of 70–80%.

*Data entry:* The researchers are blinded to the participants’ responses since the ID code is used during data entry. The woman’s self-reported data will be registered 2 hours after birth (touch screen), and 3 and 12 months after birth (web questionnaire or paper questionnaire). For details see Additional file 2. Data from the medical record will be entered by a research midwife in a web-based form. These data will be double checked. Of the four parts of the primary composite outcomes, the only one that could potentially be swayed is postpartum haemorrhage. To guard against any possibility of bias the following routines are established:

- Blood loss will be measured, not estimated
- The same team of midwives will care for women in both arms of the study and for all other women not in the study, so a biased individual will have little opportunity to care for women in the new room very often
- At least two healthcare personnel will be present at any birth, so both would need to be willing to falsify the results for this to happen, which is unlikely to occur with any frequency
- The researchers make checks on all data and will be on the lookout for any potential anomalies

All electronic data will be kept on a password-protected computer in a locked office at the labour ward for the study. All hard copies of participant details will be kept in a fireproof, locked cabinet for research data at the University of Gothenburg. All data will be stored for 10 years after the end of the study and then securely destroyed.

The healthcare costs related to the hospital stay will be collected from the hospital administrative records. As the study population constitutes only nulliparous women at term without severe increased risks, we assume that adjustments for comorbidity are not needed. A schedule of enrollment, interventions and assessments is presented in Table 2.

**Table 2:**
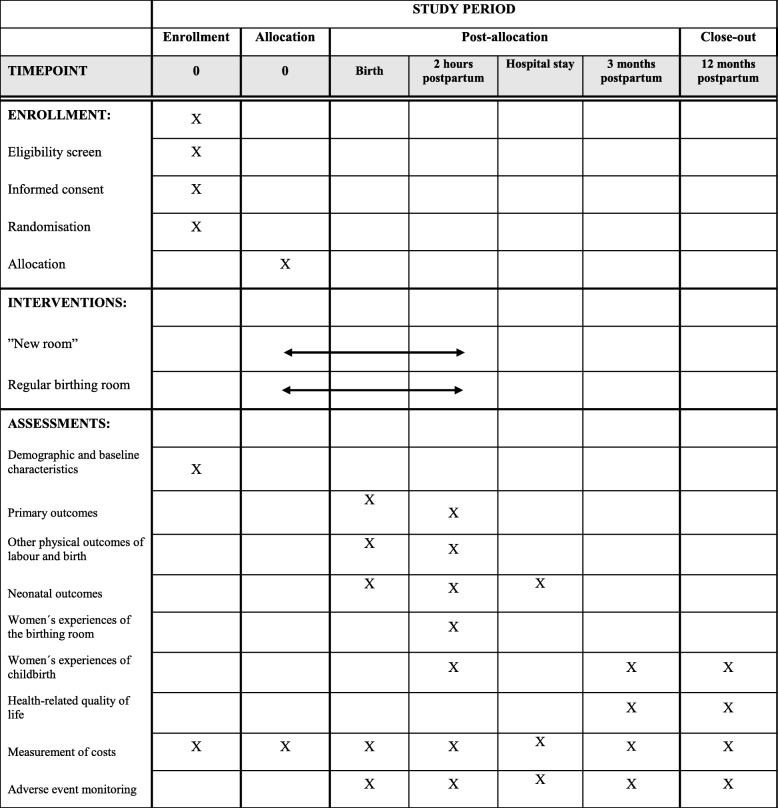
Schedule of enrollment, interventions and assessments

*Sample size*: A total of 1274 women are required (637 per group) to detect a reasonable clinical difference in the primary composite outcome of 8% between the groups (45% in control vs 53% intervention), with two-sided Fisher’s test, 80% power and 5% significance level using the ‘intention to treat’ population. To allow for 10% attrition 1401 women are required. This sample size calculation is based on data from the labour ward for the target group (Robson 1) in the year 2017 in which the three first parts in the composite score were fulfilled in 47.9% of the target group. Among these, based on a national register study [56] as no data were available from SUH, we assumed that 94% could have a positive ‘overall childbirth experience’. This implies that 0.479 × 0.94 = 0.450 = 45% fulfils all four parts in the main composite outcome. Data from the Swedish Pregnancy Register show that 84.1% of all registered women had a positive childbirth experience in 2015–2016 [56].

### Statistical methods

All main analyses will be performed on the intention to treat population and followed according to randomisation. The primary analysis and selected secondary efficacy analyses will also be performed on the ‘per protocol’ population. For comparison between the two randomised groups Fisher’s non-parametric permutation test will be used for continuous variables, Mantel-Haenszel Chi^2^ test for ordered categorical variables and Fisher’s exact test for dichotomous variables and Chi^2^ test for non-ordered categorical variables. For dichotomous outcome variables two-sided 95% confidence intervals (CIs) for the difference in proportions between groups will be calculated as well as risk ratios (RRs) with 95% CI. For continuous outcomes two-sided 95% CI for the difference in means between groups will be calculated based on Fisher’s non-parametric permutation test. The main analysis will be the above unadjusted analyses.

We will perform our primary analysis with the composite primary outcome. If group imbalances in important baseline characteristics are found, complementary analyses adjusted for these variables will be performed with analyses of covariance for continuous variables and with generalised linear models with binomial distribution and log link function in order to calculate adjusted RRs with 95% CI for dichotomous variables. The distribution of the variables will be given as mean, standard deviation (SD), median quartiles 1 and 3 (Q1, Q3) for continuous variables and as numbers and percentages for categorical variables. Imputations for missing data will be performed, when applicable. Missing data will be imputed using fully conditional multiple imputation in the main analysis. A sensitivity analysis will be performed with the full analysis dataset without imputation. All tests will be two-sided and conducted at the 5% significance level.

### The ethnographic study

An ethnographic study with participant observations documented by field notes [57] will be conducted in both types of birthing room during day, evening and night shifts. This study will explore the influence and meaning of the birthing rooms on women giving birth and thus increase the understanding of how healthcare contexts and human interactions can affect birth outcomes. It also takes an outsider perspective when interpreting how environments and cultures affect us, such as how a birthing room influences the birthing women.

The exact number of observations will be determined by data saturation, i.e. when sufficient variation and quality of data to answer the study aim are achieved. During the observations, there will be short, clarifying talks with the observed women. Field notes will also contain the observing researcher’s reflections. Based on preliminary analysis of observations, clarifying in-depth interviews will be conducted (around 6 weeks after birth) with some of the women at a place chosen by the woman. Field notes of observation, reflection notes, recorded spontaneous talks and follow-up interviews will be transcribed verbatim for analysis. The goal for the data analysis will be to explore and describe the influence of the birthing environment on the women giving birth in the two types of room. Data will be analysed using interpretation of the women’s experiences, conceptions, imaginings and practices in the birthing context in relation to safety, familiarity and choice. To ensure validity, further analyses of primary interpretations will be conducted by the responsible researcher in collaboration with the research team through recurrent discussions. This process is guided by following the hermeneutic spiral movement between the whole, the parts and a new whole described in categories and themes [58].

### Roles and responsibilities

The research team consists of the Steering Committee (Marie Berg, Lisa Goldkuhl, Christina Nilsson Helle Wijk and Cecily Begley), who are responsible for the quality and conduct of the study, and the wider members of the team (Steering Committee together with Hanna Gyllensten, Göran Lindahl, Kerstin Uvnäs Moberg). Given the intervention (use of a different type of birthing room, where care in labour is unchanged), no adverse events are envisaged. However, a Data and Safety Monitoring Committee (DSMC) will be established for the project, with clinicians, a service user representative and a statistician. Information leaflets for women include details of how to make complaints or report adverse incidents. Any such incidents reported to the team will be passed on to the DSMC for consideration. This group will be provided with an account of project progress annually, and will be contacted if there are any complaints or adverse incidents attributed to the trial. They will conduct an objective interim analysis on data from the first half of the trial, to assess for any possible harms, and will provide an outside opinion on the safety of the intervention at the end of the trial. We have not set any stopping guidelines for futility as it is important to continue to full recruitment in order to have sufficient numbers to assess results in all groups.

## Discussion

### Significance and scientific novelty of the study

Our study helps to fill existing knowledge gaps, which we have summarised in the following:

- Giving birth influences a woman and the baby for the rest of their lives [59]. The birthing environment can negatively or positively affect maternal and neonatal morbidity and decisions on future pregnancies [45] and is therefore of great societal and scientific importance.
- Despite considerable investments in renovation/construction of maternity care facilities, there is little evidence available on what is the best design of buildings and birthing environments to promote high quality care in terms of safety, including medical safety, emotional trust, integrity, privacy, familiarity and choice
- This study will provide additional evidence to guide the design of birthing rooms that contribute to optimal quality of hospital-based care at birth. Specifically it will extend the evidence-based knowledge on the design of birthing rooms and their influence on labour, birth and childbirth experiences in nulliparous women with spontaneous labour start.
- The use of an RCT to test the rooms is a quite new approach in this field, which usually uses social science methodology. Only one pilot study conducted in Canada has been published earlier [46] and two studies are ongoing, in Denmark [47] and Germany [48].
- The use of a qualitative ethnographic study will deepen understanding of the interaction between the woman and the room. It may also identify underlying factors that explain the effect of the different rooms in the RCT.
- The adaptable person-centred intervention room (new room) is expected to improve outcomes of labour and birth, including an increase in normal healthy births, a decrease in the need for augmentation during birth and an all-around better experience of the birthing process for mother and child. This is beneficial for the health, wellbeing and quality of life for the woman and her family.
- Direct economic gains to the healthcare system are expected due to the expected decreased needs for medical interventions. However, this needs to be ascertained while conducting the study. Such costs need to be compared with the costs for constructing the new room, to guide healthcare decision-makers regarding efficient use of funds when procuring design, construction and refurbishing of birthing wards. There may also be economic gains due to fewer mental, emotional and physical complications that need to be further explored in future studies.
- The results of this study can immediately be transferred into clinical practise. If the intervention birthing room results in a positive effect, the tested design of the adaptable person-centred birthing rooms can be applied in the construction of a planned new maternity care unit at the actual study hospital. Furthermore, it can guide design of birthing rooms and maternity units both across the country and internationally.

## Trial status

The study was retrospectively registered at ClinicalTrials.gov—registration date 13th of May 2019. This is the first study protocol, dated 19th June 2019. Recruitment and randomisation of participants in the study started January 2019. Inclusion of new participants is expected to be finalised by 31st December 2022 and data collection will be completed December 2023.

## Project organisation

This multi-disciplinary scientific project is conducted in collaboration with SUH; University of Gothenburg (UGOT)—Institute of Health and Care Sciences (IHCS) at Sahlgrenska Academy (SA); Chalmers University of Technology (CUT)—Centre for Healthcare Architecture (CVA); University of Borås (UB)—Academy of Health, Work and Welfare; Swedish University of Agriculture (SUA); Trinity College Dublin (TCD)—School of Nursing and Midwifery Ireland, and in joint discussion with the lay person association “Födelsehuset”.

## Supplementary information

**Additional file 1.** SPIRIT 2013 checklist: Recommended items to address in a clinical trial protocol and related documents.

**Additional file 2.** Room4Birth study variables.

## Data Availability

No one outside of the research team and the DSMC members will have access to the final trial dataset, as we have no ethical approval for this.

## Abbreviations

BMI: Body mass index
CEQ: Childbirth Experience Questionnaire
CI: Confidence intervals
CS: Caesarean section
EQ-5D: EuroQol 5 Dimension health state questionnaire
FOBS: Fear of Birth Scale
Q1: Quartile 1
Q3: Quartile 3
RCT: Randomised controlled study
RR: Risk ratio
SD: Standard deviation
SUH: Sahlgrenska University Hospital
