# Supplementary material for: Room4Birth - the effect of an adaptable birthing room on labour and birth outcomes for nulliparous women at term with spontaneous labour start: study protocol for a randomised controlled superiority trial in Sweden
Source: Trials. 2019 Nov 19;20:629. doi: 10.1186/s13063-019-3765-x (PMC6862754; doi:10.1186/s13063-019-3765-x)
Supplement: Supplementary file 2 — Additional file 2. Room4Birth study variables. [file 13063_2019_3765_MOESM2_ESM.doc]

**Room4Birth Study variables**

**QUESTIONS ANSWERD ON A TOUCH SCREEN BY THE WOMAN 2 HOURS POST PARTUM**

| **VARIABLES** | **VARIABLE VALUES** |
| --- | --- |
| ID | 1000-6000 (answered by staff) |
| What type of room was the woman allocated to? | 1) Test room 2) Regular room (answered by staff) |
| Age | 18-60 |
| Education | 1) Compulsory / elementary school (year 1-9 or equivalent)  2) High school (year 10-12 or equivalent)  3) University or College |
| What is your current family situation? | 1) Cohabiting with the baby’s father  2) Single  3) Other family situation |
| In which country were you born? | 1) Sweden  2) Other country |
| Which year did you come to Sweden? | Figures |
| Have you participated in a childbirth preparation course or taken part in information about labour? | 1. Childbirth preparation course (for example ”Magplasket”, ”Föda Utan Rädsla”, Pregnant yoga or in any other childbirth preparation course) 2. Information at the hospital Östra Sjukhuset 3. Have not participated in any of the above   *(you can choose several alternatives)* |
| Who was with you during labour apart from the health professionals? | 1. Partner 2. Doula 3. Other person 4. Nobody else was with me   *(you can choose several alternatives)* |
| How was your overall childbirth experience? | (Indicate your opinion by marking on the line between the two end-points)  from very bad to very good |
| How do you rate worry when you THINK ABOUT YOUR COMPLETED LABOUR AND BIRTH? | (Indicate your opinion by marking on the line between the two end-points)  *from calm to worried* |
| How do you rate fear when you THINK ABOUT YOUR COMPLETED LABOUR AND BIRTH? | (Indicate your opinion by marking on the line between the two end-points)  *from no fear to strong fear* |
| How do you feel right now ABOUT GIVING BIRTH AGAIN? | (Indicate your opinion by marking on the line between the two end-points)  *from calm to worried* |
| How do you feel right now ABOUT GIVING BIRTH AGAIN? | (Indicate your opinion by marking on the line between the two end-points)  *from no fear to strong fear* |
| To what extent did the room design contribute to your sense of safety? | 1) To a very high degree  2) To a high degree  3) To a low degree  4) Not at all |
| How important is this for you? | 1) Very important  2) Important  3) Slightly important  4) Not important |
| To what extent did the room design contribute to your sense of control? | 1) To a very high degree  2) To a high degree  3) To a low degree  4) Not at all |
| How important is this for you? | 1) Very important  2) Important  3) Slightly important  4) Not important |
| To what extent did the room design contribute to your sense of integrity? | 1) To a very high degree  2) To a high degree  3) To a low degree  4) Not at all |
| How important is this for you? | 1) Very important  2) Important  3) Slightly important  4) Not important |
| To what extent did the room design adapt to your needs and requests? | 1) To a very high degree  2) To a high degree  3) To a low degree  4) Not at all |
| How important is this for you? | 1) Very important  2) Important  3) Slightly important  4) Not important |
| Were the functions in the birthing room meaningful for your birth? | 1) To a very high degree  2) To a high degree  3) To a low degree  4) Not at all |
| How important is this for you? | 1) Very important  2) Important  3) Slightly important  4) Not important |
| Rank from 1 to 9 the following functions in the birthing room regarding their importance in relation to the childbirth.  1 is the most important and 9 is the least important.  (this question is only for women allocated to the test room) | 1. Curtain from hallway to room 2. Sofa 3. Adjustable chair for partner 4. Bathtub 5. Lighting, general in the room 6. Lighting, dimmable 7. The projection on the two walls with associated light and sound 8. Medical technology covered with wood panel and bedspread 9. Birth support rope |

**VARIABLES TO COLLECT FROM MEDICAL JOURNALS**

| **VARIABLES** | **VARIABLE VALUES** |
| --- | --- |
| BMI | Figures with two decimals |
| Mental illness treatment | Yes/No |
| Seeking help for fear of childbirth | Yes/No |
| Contractions starting | Date |
| Contractions starting | Time |
| Regular contractions | Date |
| Regular contractions | Time |
| Rupture of membranes | Date |
| Rupture of membranes | Time |
| Amniotomy | Yes/No |
| Amniotomy | Date |
| Amniotomy | Time |
| Use of epidural anaesthesia | Yes/No |
| Use of epidural anaesthesia | Date and time |
| Intrapartum fever | Yes/No |
| Use of bathtub | Yes/No |
| Use of synthetic oxytocin for augmentation of labour | Yes/No |
| Oxytocin infusion starting | Date, Time |
| Oxytocin infusion, stage of labour | Active stage/second stage of labour/Pushing stage/ post partum |
| Oxytocin infusion ends | Date, Time |
| Highest dose of synthetic Oxytocin | ml/h |
| Pushing contractions | Date, Time |
| Episiotomy | Yes/No |
| Tear clitoris/labia | Yes/No |
| Vaginal tear | Yes/No |
| Perineal Tear grade 1 and 2 | Yes/No |
| Perineal Tear grade 3 | Yes/No |
| Perineal Tear grade 4 | Yes/No |
| Cervix Tear | Yes/No |
| Deep vaginal tear that need operative care | Yes/No |
| Time of care: from enrollment to birth | Hours and minutes |
| Time of care: from randomisation to birth | Hours and minutes |
| Post partum blood loss total | ml |
| Post partum blood loss total > 500 ml | n Yes/No |
| Post partum haemorrhage total > 1000 ml | n Yes/No |
| manual removal of placenta | Yes/No |
| Mode of birth | n, Spontaneous vaginal (=1)  Vacuum extraction (=2)  Caesarean section (=3) |
| Indication for section | None/ prolonged labour/ birth asphyxia/birth contract |
| Indication VE | None/ prolonged labour/ birth asphyxia |
| Birth position of woman | Free text |
| Gestational week at birth | Weeks, days |
| Apgar score at 5 minutes | 0-10 |
| Apgar Score < 7 (0-6) at 5 minutes | Yes/No |
| Apgar Score < 4 (1-3) at 5 minutes | Yes/No |
| Stillborn | Yes/No |
| Birth weight | grams |
| Sex of neonate | Flicka, Pojke |
| Ph in umbilical artery | figures |
| CO2 in umbilical artery | figures |
| O2 in umbilical artery | figures |
| BE in umbilical artery | figures |
| Ph in umbilical vein | figures |
| CO2 in umbilical vein | figures |
| O2 in umbilical vein | figures |
| BE in umbilical vein | figures |
| Skin to skin contact within the first hour | Yes/No |
| Breast feeding within the first 2 hours | Yes/No |
| neonatal death during hospital stay | Yes/No |
| Admission to the neonatal unit | Yes/No |
| Time of care from enrollment to randomisation | Hours, minutes |
| Total time of care at the maternity ward (arrival at the maternity ward to leaving the hospital) | Hours, minutes |
| Total time of care at the hospital (from arrival to the labour ward to leaving the hospital) | Hours, minutes |
| Total time of care at the hospital, woman | Hours, minutes |
| Total time of care at the hospital, neonate | Hours, minutes |

**WEB QUESTIONNAIRE 3 AND 12 MONTHS POST PARTUM SENT BY E-MAIL**

| **VARIABLES** | **VARIABLE VALUES** |
| --- | --- |
| How was your overall childbirth experience? | (Indicate your opinion by marking on the line between the two end-points)  Very bad – very good |
| I would have preferred another form of pain relief | - Totally agree - Mostly agree - Mostly disagree - Totally disagree |
| I could get up and move around as much as I wanted | - Totally agree - Mostly agree - Mostly disagree - Totally disagree |
| I could give birth in the way I wanted | - Totally agree - Mostly agree - Mostly disagree - Totally disagree |
| **CEQ: CHILDBIRTH EXPERIENCE QUESTIONNAIRE** | |
| Labour and birth went as I had expected  (Please tick the box below the response choice that best corresponds to your opinion) | - Totally agree - Mostly agree - Mostly disagree - Totally disagree |
| I felt strong during labour and birth | - Totally agree - Mostly agree - Mostly disagree - Totally disagree |
| I felt scared during labour and birth | - Totally agree - Mostly agree - Mostly disagree - Totally disagree |
| I felt capable during labour and birth | - Totally agree - Mostly agree - Mostly disagree - Totally disagree |
| I was tired during labour and birth | - Totally agree - Mostly agree - Mostly disagree - Totally disagree |
| I felt happy during labour and birth | - Totally agree - Mostly agree - Mostly disagree - Totally disagree |
| I felt that I handled the situation well | - Totally agree - Mostly agree - Mostly disagree - Totally disagree |
| I wish the staff had listened to me more during labour and birth | - Totally agree - Mostly agree - Mostly disagree - Totally disagree |
| I took part in decisions regarding my care and treatment as much as I wanted | - Totally agree - Mostly agree - Mostly disagree - Totally disagree |
| Both my partner and I were treated with warmth and respect | - Totally agree - Mostly agree - Mostly disagree - Totally disagree |
| I received the information I needed during labour and birth | - Totally agree - Mostly agree - Mostly disagree - Totally disagree |
| I would have preferred the midwife to be more present during labour and birth | - Totally agree - Mostly agree - Mostly disagree - Totally disagree |
| I would have preferred more encouragement from the midwife | - Totally agree - Mostly agree - Mostly disagree - Totally disagree |
| The midwife conveyed an atmosphere of calm | - Totally agree - Mostly agree - Mostly disagree - Totally disagree |
| The midwife helped me to find my inner strength | - Totally agree - Mostly agree - Mostly disagree - Totally disagree |
| My impression of the team’s medical skills made me feel secure | - Totally agree - Mostly agree - Mostly disagree - Totally disagree |
| I have many positive memories from childbirth | - Totally agree - Mostly agree - Mostly disagree - Totally disagree |
| I have many negative memories from childbirth | - Totally agree - Mostly agree - Mostly disagree - Totally disagree |
| Some of my memories from childbirth make me feel depressed | - Totally agree - Mostly agree - Mostly disagree - Totally disagree |
| As a whole, how painful did you feel childbirth was? | (Indicate your opinion by marking on the line between the two end-points)  From no pain to worst imaginable pain |
| As a whole, how much control did you feel you had during childbirth? | (Indicate your opinion by marking on the line between the two end-points)  From No control to complete control |
| As a whole, how secure did you feel during childbirth? | (Indicate your opinion by marking on the line between the two end-points)  Not at all secure to completely secure |
| Additional comments |  |
| **FOBS: FEAR OF BIRTH SCALE AND, FEAR OF GIVING BIRTH AGAIN** | |
| How do you rate worry when you think about your completed labour and birth? | (Indicate your opinion by marking on the line between the two end-points)  From calm to worried |
| How do you rate fear when you think about your completed labour and birth? | (Indicate your opinion by marking on the line between the two end-points)  From strong fear to no fear |
| How do you rate worry when you think about giving birth again? | (Indicate your opinion by marking on the line between the two end-points)  From calm to worried |
| How do you rate fear when you think about giving birth again? | (Indicate your opinion by marking on the line between the two end-points)  From strong fear to no fear |
| **EQ-5D: EUROQOL FIVE DIMENSION** | |
| Mobility  (Pease tick the ONE box that best describes your health TODAY) | - I have no problems in walking about - have some problems in walking about - I am confined to bed |
| Self-Care | - I have no problems with self-care - I have some problems washing or dressing myself - I am unable to wash or dress myself |
| Usual Activities *(e.g. work, study, housework, family or leisure activities)* | - I have no problems with performing my usual activities - I have some problems with performing my usual activities - I am unable to perform my usual activities |
| Pain / Discomfort | - I have no pain or discomfort - I have moderate pain or discomfort - I have extreme pain or discomfort |
| Anxiety / Depression | - I am not anxious or depressed - I am moderately anxious or depressed - I am extremely anxious or depressed |
| YOUR OWN HEALTH STATE TODAY | (Mark on this thermometer, where 100 is the best imaginable health state and 0 is the worst imaginable health state) |
